# Establishing Reliable Cu-64 Production Process: From Target Plating to Molecular Specific Tumor Micro-PET Imaging

**DOI:** 10.3390/molecules22040641

**Published:** 2017-04-17

**Authors:** Qinghua Xie, Hua Zhu, Feng Wang, Xiangxi Meng, Qiushi Ren, Chuanqin Xia, Zhi Yang

**Affiliations:** 1 College of Chemistry, Sichuan University, Chengdu 610064, China; qinghxie@163.com; 2 Key Laboratory of Carcinogenesis and Translational Research (Ministry of Education), Department of Nuclear Medicine, Peking University Cancer Hospital & Institute, Beijing 100142, China; zhuhuananjing@163.com (H.Z.); windtigerwf@163.com (F.W.); 3 Department of Biomedical Engineering, College of Engineering, Peking University, Beijing 100871, China; mengxiangxi@pku.edu.cn (X.M.); renqsh@coe.pku.edu.cn (Q.R.)

**Keywords:** copper-64, solid target, Rituximab, positron emission tomography (PET)

## Abstract

Copper-64 is a useful radioisotope for positron emission tomography (PET). Due to the wide range of applications, the demand of ^64^Cu with low metallic impurities is increasing. Here we report a simple method for the efficient production of high specific activity ^64^Cu using a cyclotron for biomedical application. We designed new equipment based on the plating of enriched ^64^Ni as the target, and used automated ion exchange chromatography to purify copper-64 efficiently after irradiation and dissolution of the target in good radiochemical and chemical yield and purity. The ^64^Cu radionuclide produced using 99.32% enriched ^64^Ni with a density of 61.4 ± 5.0 mg/cm^2^, reaching a total radioactivity greater than 200 mCi, with specific activity up to 5.6 GBq/μmoL. It was further incorporated into modified monoclonal antibody DOTA-rituximab to synthesize ^64^Cu-DOTA-rituximab, which was used successfully for micro-PET imaging.

## 1. Introduction

Copper-64 (^64^Cu) is an attractive radionuclide of considerable interest for positron emission tomography (PET) imaging and radiotherapy due to its intrinsic physical and chemical properties. It has high spatial resolution comparable to ^18^F radionuclide, with comparable average free travel distance for their generated positrons (R_ave._(β^+^) = 0.70 and 0.69 mm, respectively), due to their comparable positron energy (0.656 MeV and 0.635 MeV, respectively) [1,2]. It has a relatively long half-life of 12.7 h, compared with fluorine-18 (*t*_1/2_ = 110 min) and carbon-11 (*t*_1/2_ = 20.4 min). In addition, ^64^Cu also emits β^−^ and Auger electrons, enabling it to be useful for both PET imaging and radiotherapy. Moreover, the versatile coordination chemistry of ^64^Cu allows for its reaction with a wide variety of chelator systems, such as DOTA, NOTA, TETA and CB-TE2A, that can be linked to antibodies, peptides and nanoparticles [3]. The ^64^Cu radioisotope can be used for the design and synthesis of a wide range of radio-probes, providing attractive candidates for PET imaging.

A number of radiotracers involving ^64^Cu as radionuclide have been applied in nuclear medicine as a means of studying their PET imaging [4,5,6]. Incorporation of ^64^Cu into diacetyl-bis(*N*^4^-methylthiosemicarbazone) (ATSM) ligand was used for PET hypoxia imaging, such as in head and neck cancer, and cardiac conditions [7,8]. More radiotracers are in clinical development [9,10,11,12], especially those with peptides and antibodies. For example, ^64^Cu-DOTA-trastuzumab was used to conduct PET imaging of HER2-positive lesions in patients with primary and metastatic breast cancer [13]. The PET image of ^64^Cu-DOTATATE provided superior image quality, and detected more lesions than ^111^In-DTPA-octreotide [14]. Grubmüller, B. et al. investigated the diagnostic potential of ^64^Cu-PSMA-617 in patients with prostate adenocarcinoma [15].

^64^Cu has been produced at many centers [16,17,18,19]. Among the nuclear reactions examined, the ^64^Ni (p, n) ^64^Cu method is the best and widely used, since high production yield of the ^64^Cu can be obtained with low energy protons in this route [20,21]. At Washington University, an effective method was investigated to produce high specific activity ^64^Cu on a small biomedical cyclotron using the ^64^Ni (p, n) ^64^Cu nuclear reaction, and ^64^Cu has been produced for more than 17 years by the irradiation of electroplated enriched ^64^Ni targets in this center [20,22]. The Turku PET Centre has been producing ^64^Cu since 2008 using ^64^Ni (p, n) ^64^Cu reaction, and also handles the irradiated target, radioactive liquids and gases using automated equipment [23,24]. At the University of Wisconsin, ^64^Cu and ^61^Co radionuclides have been simultaneously produced using the ^64^Ni (p, n) ^64^Cu nuclear reaction on a low energy proton-only cyclotron [25]. Ohya, T. et al. (2016) produced high-quality ^64^Cu for routine use via an electrodeposited ^64^Ni target, and successfully reduced the metallic impurities level of the ^64^Cu product, such as Co and Ni [21]. Other nuclear reactions examined include ^64^Ni (d, 2n) ^64^Cu, ^64^Zn (d, 2p) ^64^Cu, ^64^Zn (n, p) ^64^Cu [26,27]. In China, researchers also have a growing interest in ^64^Cu, and the demand of no-carrier added ^64^Cu has started to increase.

Here we report a robust, reliable and user-friendly plating vessel, which can be used for the effective preparation of the ^64^Ni solid target. The production of ^64^Cu was performed on a Sumitomo HM-20 biomedical cyclotron (20 MeV) via the ^64^Ni (p, n) ^64^Cu reaction. The γ-ray spectroscopy of the produced ^64^Cu solution was also measured to evaluate the radionuclide impurities. After ^64^Cu was purified, a labeling experiment to synthesize ^64^Cu-DOTA-Rituximab was performed to examine the quality of ^64^Cu, including labeling yield and radiochemical purity of the radiotracer, which targets the CD20 antigen, which is expressed on B cell lymphocytes and in the majority of non-Hodgkin’s lymphoma (NHL) [28]. The synthesized radiotracer was further examined by micro-PET imaging using SCID (severe combined immune deficiency) mice bearing Ramos RA1 tumors which overexpress CD20 antigen.

## 2. Results and Discussion

### 2.1. Preparation of ^64^Ni Target

**Figure 1:**
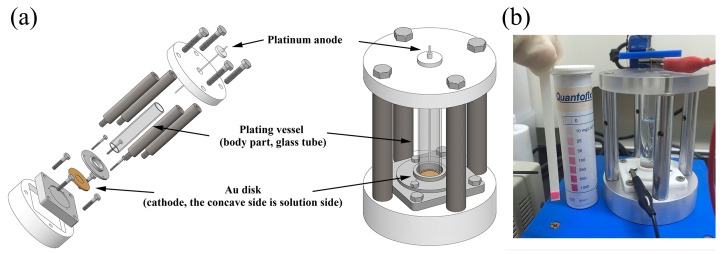
The ^64^Ni plating vessel. (**a**) Illustration of new ^64^Ni plating vessel by scheme; (**b**) Picture of the actual electric plating unit.

In order to make ^64^Cu via the nuclear reaction of ^64^Ni (p, n) ^64^Cu, we made the enriched ^64^Ni targets on a gold (Au) disk by electrodeposition of ^64^Ni from an aqueous solution of Ni(NH_3_)_6_^2+^ at pH = 9.05, using a robust, reliable and user-friendly apparatus (Figure 1). The Au disk (30.8 mm diameter × 1.5 mm thickness) was used as a cathode, and a platinum wire was used as an anode. A cavity of 12.1 mm diameter and 0.2 mm depth was milled into the Au disk and ^64^Ni (99.32%) was plated into this cavity. The ^64^Ni electroplating was performed with a constant current of 15–25 mA at 2.4–2.6 V in the aqueous solution for 48–72 h. During the electrodeposition, the green color of the ^64^Ni plating solution gradually faded away and the bubbling of H_2_ gas was clearly visible. When the ^64^Ni plating solution became colorless, the electrodeposition was finished. The absence of Ni^2+^ was also confirmed by analytical test strips. After cleaning and drying, the plated ^64^Ni on Au-disk was 70.6 ± 5.8 mg and the density of the plated ^64^Ni was 61.4 ± 5.0 mg/cm^2^, assuming a uniform thickness on the disk.

### 2.2. Quality Control of ^64^Ni Target

**Figure 2:**
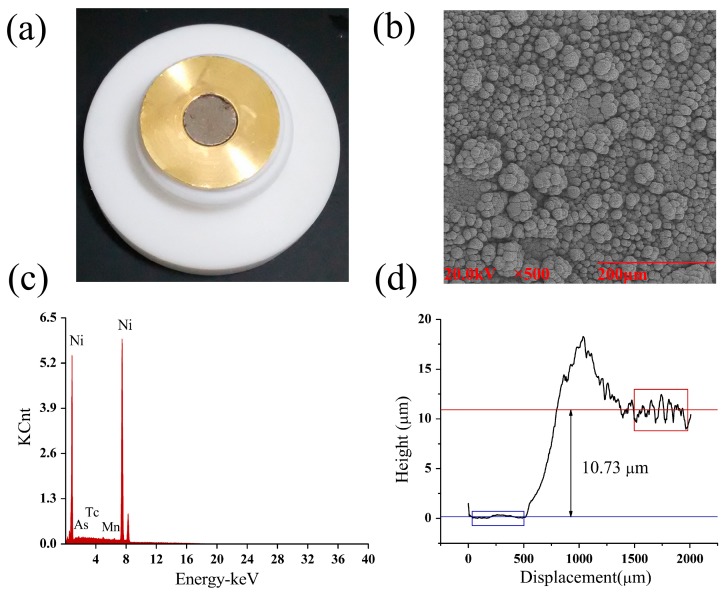
The ^64^Ni target produced in this study. (**a**) Photo of the ^64^Ni target; (**b**) The SEM image of the ^64^Ni target; (**c**) The EDS spectrum of the ^64^Ni target; (**d**) The thickness measurement of the ^64^Ni solid target.

The quality of the ^64^Ni solid target prepared above was evaluated by a number of physical techniques, to determine the uniformity, to characterize the metallic impurities, and to measure the thickness of ^64^Ni layer on Au-disk. The SEM (scanning electron microscopy) image (Figure 2b) of the ^64^Ni solid target showed the uniform layer of Ni on Au surface. The EDS (energy dispersive X-ray spectroscopy) (Table 1 and Figure 2c) results showed no significant amount of metallic impurities in the ^64^Ni layer. The ^64^Ni target thickness on Au-disk was measured to be 10.73 μm by alpha step apparatus (Figure 2d).

### 2.3. Preparation of ^64^Cu

After irradiation of 5 h, the ^64^Ni target was dissolved in 6 M hydrochloride acid, and then the solution was load to an anion exchange column to separate into different components. The ^64^Ni was washed out with 6 M HCl and collected for recycling. Due to the elevated cost of enriched ^64^Ni, recycling of the target material for re-use could reduce the production cost of ^64^Cu, without sacrificing the quality of subsequent ^64^Cu production. When the eluted was switched to 1 M HCl, the first band coming out was co-produced cobalt radioisotopes (approximately 1 mL), and the second was the ^64^Cu, which was collected and evaporated to dryness. The residue was dissolved in 0.1 M HCl for further use. The separation process of ^64^Cu took about 2.5 h after irradiation.

### 2.4. Quality of ^64^Cu

**Figure 3:**
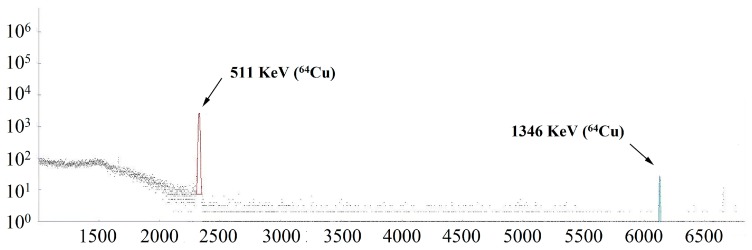
Gamma spectra of ^64^CuCl_2_ solution after purification.

**Figure 4:**
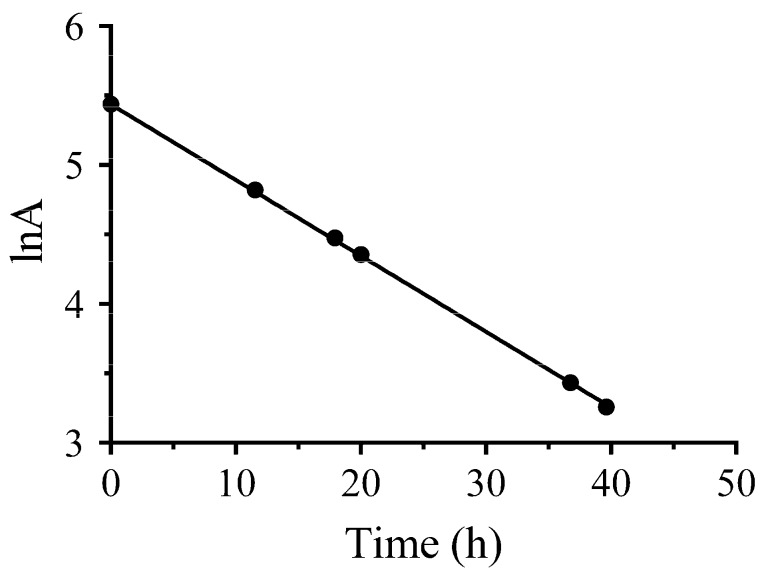
The radioactivity of the produced ^64^Cu measured at different time points vs. time, with fitted equation, on a logarithmic scale.

The quality of ^64^Cu produced was evaluated by analysis of its metallic impurities and measurement of half-life of its radioactivity. The inductively coupled plasma-mass spectrometry (ICP-MS) analysis was used to evaluate the amount of metallic impurities in a decayed ^64^Cu solution, and showed a concentration of 6.339 ppb of Ni (0.127 μg), 4.112 ppb of Cu (0.082 μg), 5.502 ppb of Zn (0.110 μg), 0.108 ppb of Fe (0.002 μg), 0.102 ppb of Co (0.002 μg), 0.669 ppb of Ga (0.013 μg). The gamma spectrum of the produced ^64^CuCl_2_ solution (Figure 3) showed that the ^64^Cu radionuclide purity was >99%. The half-life of the produced radioactivity was determined by the radioactivity measured at different time points into the following equations:

(1) $$A=A_{0}e^{-\lambda t},\;\mathrm{lnA}=-\lambda t+{\mathrm{lnA}}_{0},\;\ln\frac{A}{A_{0}}=-\lambda t$$

where A is the radioactivity of the ^64^Cu at time t, A_0_ is the radioactivity of the ^64^Cu at 0 h, and λ represents the constant. We obtained the following equation (Figure 4):

(2) lnA=−0.05456t+5.438

when $t=T_{1/2}$, the $A=\frac{1}{2}A_{0}$

(3) $$\ln\frac{1}{2}=-0.05456T_{1/2}$$

The half-life of the produced ^64^Cu was calculated:

$$T_{1/2}=\frac{\ln2}{0.05456}=12.704$$

The half-life calculated ($T_{1/2}=12.704$ h) of the produced ^64^Cu is in accordance with that of the radioisotope ^64^Cu ($T_{1/2}=12.7$ h).

### 2.5. Radio-Synthesis of ^64^Cu-DOTA-Rituximab and Micro-PET Imaging

**Figure 5:**
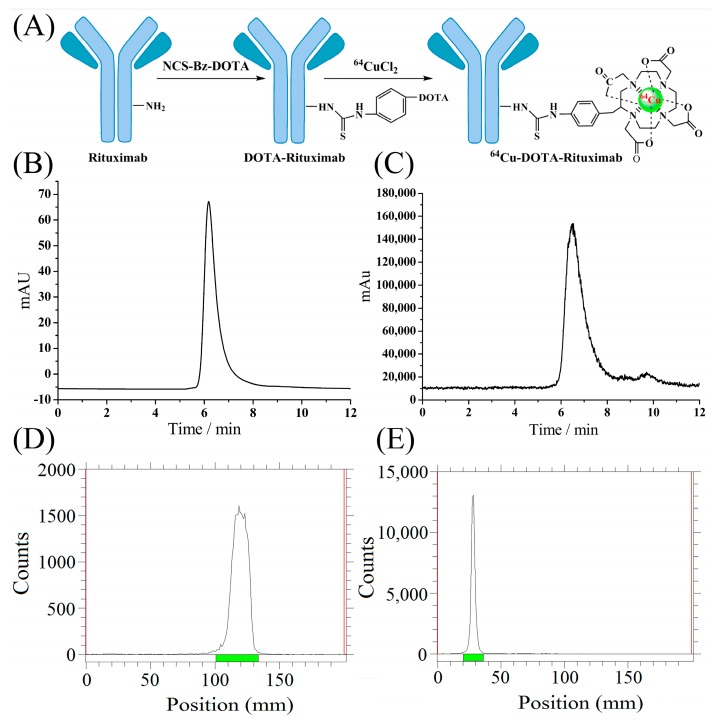
Radio-synthesis of ^64^Cu-DOTA-rituximab. (**A**) Modification of Rituximab and radiolabeling by ^64^Cu radionuclide; (**B**) The Radio-HPLC (radioactive high performance liquid chromatography) chromatograph of rituximab; (**C**) The Radio-HPLC chromatograph of ^64^Cu-DOTA-rituximab after purification by PD-10 column; (**D**) The Radio-TLC (radioactive thin-layer chromatography) image of ^64^CuCl_2_; (**E**) The Radio-TLC image of ^64^Cu-DOTA-rituximab after purification by PD-10 column.

**Figure 6:**
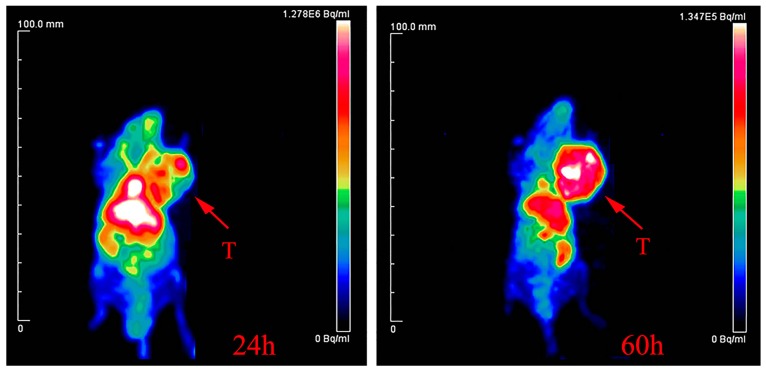
Micro-PET image of ^64^Cu-DOTA-rituximab in SCID mice bearing Ramos RA1 tumors at 24 h and 60 h post-intravenous injection. The arrows indicate the location of tumor.

To assess the quality and quantity of the produced ^64^Cu, we made ^64^Cu-DOTA-rituximab (Figure 5), with high chemical yield, high radiochemical purity, and high specific activity. Micro-PET imaging of the radiotracer in mice bearing Ramos RA1 tumors clearly showed the tumor at 24 h and 60 h post-injection, with excellent resolution and clarity at the latter (Figure 6).

## 3. Materials and Methods

### 3.1. Materials and Reagents

High purity reagents were used for production of ^64^Cu in this study. Isotopically enriched ^64^Ni (99.32% ^64^Ni; 0.13% ^58^Ni; 0.07% ^60^Ni; 0.01% ^61^Ni; 0.47% ^62^Ni) was from Isoflex Company (San Francisco, CA, USA); (NH_4_)_2_SO_4_ (99.999% metals basis) from Alfa Aesar (Ward Hill, MA, USA); concentrated HCl (99.999% trace metals basis), HNO_3_ (99.999% trace metals basis), and NH_4_OH (99.99% trace metals basis) were purchased from Sigma-Aldrich (St. Louis, MO, USA); AG^®^1-X8 ion exchange resin was from Bio-Rad Laboratories (Hercules, CA, USA); platinum wire (99.997% metals basis) from Alfa Aesar (Ward Hill, MA, USA); Ni^2+^ analytical test strips from Qtantofix (Sigma-Aldrich, St. Louis, MO, USA); disposable PD-10 desalting columns were from GE Healthcare (Piscataway, NJ, USA).

### 3.2. Equipment

The alpha step apparatus (Alpha-step IQ, KLA-Tencor, Milpitas, CA, USA), scanning electron microscopy with energy dispersive X-ray spectroscopy (SEM-EDS) (1910FE, AMRAY, Pawtucket, RI, USA) were used to characterize the quality of the ^64^Ni solid target produced in this study. The irradiation experiments were performed using a Sumitomo HM-20 cyclotron (20 MeV, Sumitomo Heavy Industries, Ltd., Tokyo, Japan). The separation of ^64^Cu was performed using a ^64^Cu separation system (Industrial Equipment Division, Sumitomo Heavy Industries, Ltd., Tokyo, Japan). Inductively coupled plasma-mass spectrometry (ICP-MS) (ELEMENT XR mass spectrometer, Thermo Fisher, Bremen, Germany) was used to analyze the purity of the ^64^Cu sample. The Agilent Technologies 1200 series of high performance liquid chromatography (HPLC) system (Agilent, Lake Forest, CA, USA) equipped with both a UV absorption detector and a B-Fc-1000 HPLC radioactivity detector (Bioscan, Washington, DC, USA) and the radioactive thin-layer chromatography scanner (Radio-TLC) (Bioscan, IAR-2000, Washington, DC, USA) were used to analyze the radiochemical purity of tracers.

### 3.3. Plating Solution

The ^64^Ni electroplating solution was prepared as reported previously with some modifications [25]. The enriched ^64^Ni metal (65–80 mg) was dissolved in 5 mL of warm 6 M HNO_3_. After the metal was completely dissolved, the solution was then evaporated to dryness under vacuum. The green-colored residue was dissolved in 300 µL of concentrated H_2_SO_4_ and the solution then diluted with 2 mL of 18 MΩ·cm water (Milli-Q Waters, Millipore Corporation, Billerica, MA, USA) slowly and carefully. The pH of the solution was adjusted to 9.05 ± 0.05 by adding about 1.5 mL concentrated NH_4_OH. To this solution, ~300 mg of (NH4)_2_SO_4_ was added and the volume of the solution was adjusted to 5 mL with 18 MΩ·cm water. The final solution was transferred to the electroplating cell for target plating.

### 3.4. Characterizations of Ni-64 Target

After electroplating, the ^64^Ni solid target was examined by measuring ^64^Ni thickness, composition and structure. The thickness was measured by alpha step apparatus, the composition was measured by scanning electron microscopy with energy dispersive X-ray spectroscopy (SEM-EDS), and the structure was characterized by scanning electron microscopy (SEM). Meanwhile, the identity of metallic impurities of the ^64^Ni target was further analyzed by energy dispersive X-ray spectroscopy (EDS).

### 3.5. Preparation of ^64^Ni Target and Irradiation

The ^64^Ni targets were prepared by electrodeposition of the enriched ^64^Ni (99.32%) solution prepared as described above. Electroplating of ^64^Ni was realized using a plating vessel of our own design and manufacture (Figure 1), where the Au disk was used as a cathode and platinum wire as an anode. The electrodeposition of ^64^Ni was achieved at 2.4–2.6 V and 15–25 mA with the platinum anode at ~1 cm from the Au disk electrode. This process took 48–72 h. The enriched ^64^Ni target was irradiated at 12.5 MeV (which decreased by Al) with 20 μA current on a Sumitomo HM-20 cyclotron (20 MeV) for about 5–7 h. ^64^Cu was produced from the ^64^Ni (p, n) ^64^Cu nuclear reaction.

### 3.6. Radiochemical Separation

**Figure 7:**
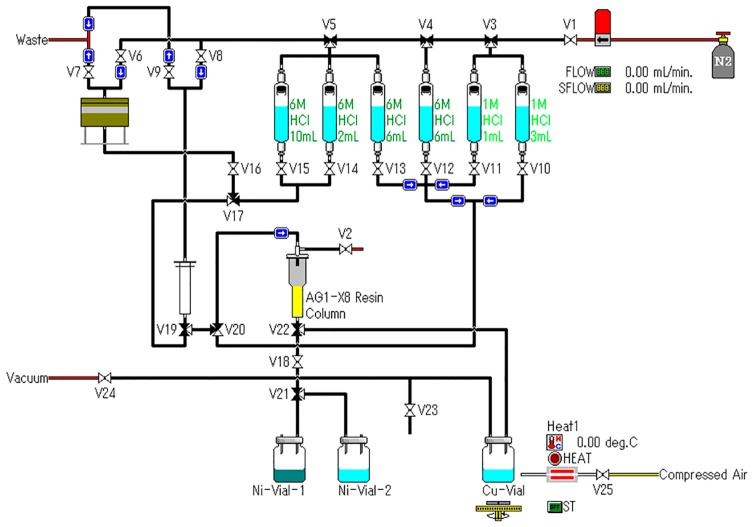
Schematic representation of the automated ^64^Cu separation system.

After irradiation, the ^64^Ni target was placed into the dissolving bath of 6 M HCl. The complete dissolution of the target material took about 40 min under heating, and then the solution was loaded on an anion exchange column (AG 1-X8) pretreated with 18 MΩ·cm water and 6 M HCl, sequentially. The enriched ^64^Ni was eluted with 6 M HCl and collected for recycling, and the ^64^Cu fraction was eluted with 1 M HCl, which was further evaporated to dryness. The residue was finally dissolved in 0.1 M HCl for further use. All of these procedures were performed using an automated system and carried out in a hot cell with remote control (Figure 7, Sumitomo Heavy Industries, Ltd., Tokyo, Japan).

### 3.7. Radio-nuclide Analysis

The radionuclide identity and purity of the produced ^64^CuCl_2_ solution were measured using γ-ray spectroscopy (HTA Co., Ltd., Beijing, China). In addition, the identity of radioactivity of the produced ^64^Cu was further confirmed by measurement of its half-life, by measuring radioactivity at different time points. A decayed sample from the produced ^64^Cu was also analyzed by inductively coupled plasma-mass spectrometry (ICP-MS) for traces of metallic impurities.

### 3.8. Radiolabeling of DOTA-Rituximab and Micro-PET Imaging

To assess the quality of the produced ^64^Cu (quantity, specific activity and purity), the synthesis of a radiotracer, ^64^Cu-DOTA-rituximab, was performed. Here, the site-specific modification of monoclonal antibody DOTA-rituximab which contains two DOTA chelator in each antibody was used as we previously reported [29]. The ^64^CuCl_2_ solution prepared above was reacted with DOTA-rituximab in a solution of pH = 5.5 at RT for 30 min. After incubation, the radiotracer was purified using a PD-10 desalting column, and characterized by Radio-TLC and Radio-HPLC, to determine the labeling yield and radiochemical purity. The identity of the radiotracer, ^64^Cu-DOTA-rituximab, was further evaluated by PET imaging, which was carried out on a micro-PET rodent model scanner as reported earlier [29]. After formulation, ^64^Cu-DOTA-rituximab (0.5 mCi) was injected intravenously into the tail vein of the mice bearing Ramos RA1 tumors (*n* = 3), which overexpress CD20 antigen, and the animals were imaged with micro-PET at both 24 and 60 h post-injection.

## 4. Conclusions

In this study, we presented the improved method for the preparation of ^64^Cu, especially the improved efficiency of electroplating of ^64^Ni. The ^64^Ni solid target on an Au-disk has a uniform surface, with the thickness of 10.73 μm, and no metallic impurities. The total plated ^64^Ni was 70.6 ± 5.8 mg and the density of the plated ^64^Ni was 61.4 ± 5.0 mg/cm^2^ (assuming a uniform thickness). After irradiation of the target and purification, the gamma spectrum of the produced ^64^Cu showed its radionuclide purity to be >99%, with both peaks at 511 keV and 1346 keV. In addition, the produced ^64^CuCl_2_ solution has high specific activity up to 5.6 GBq/μmoL. It was further incorporated into the modified monoclonal antibody DOTA-rituximab to synthesize ^64^Cu-DOTA-rituximab, which was further used successfully for micro-PET imaging.

## Figures and Tables

**Table 1:** The metallic impurities in the plated ^64^Ni target, analyzed by EDS.

Element	Wt %	At %
AsL	00.00	00.00
TcL	00.15	00.09
MnK	00.07	00.07
NiK	99.78	99.84
